# Microbe Power!

**DOI:** 10.1289/ehp.113-a754

**Published:** 2005-11

**Authors:** David C. Holzman

Increasingly, problems of rising energy demands, dwindling resources, and pollution concerns are being mitigated by turning waste into usable products. Now some researchers are eyeing organic wastes from homes, food processing, and other sources as an energy feedstock—bacteria including *Rhodoferax* and *Geobacter* are being harnessed in devices called microbial fuel cells (MFCs) to break down organic waste products, converting the energy of their chemical bonds into electricity and hydrogen.

## Significant Energy Resource

In the United States, 46 trillion liters of household wastewater are treated annually, according to an article by Bruce Logan, director of the Hydrogen Energy Center at The Pennsylvania State University, in the 1 May 2004 issue of *Environmental Science & Technology*. This costs $25 billion, and the electricity required—mostly for aeration—constitutes 1.5% of the electricity used in the nation, says Lars Angenent, an assistant professor in the Department of Chemical Engineering at Washington University in St. Louis. According to Angenent, most of that energy could be saved by treating wastewater using MFCs. He says one of these devices could produce enough extra energy to power 900 homes by treating the wastes from a single large food processing plant. According to Logan, MFCs would cut the cost of aerating activated sludge in wastewater by as much as 50% of the electricity usage, and should generate 50–90% less solids to be disposed of.

Logan put this potential in context in his 1 May 2004 article when he wrote that the United States consumed 97 quads (short for “quadrillion British thermal units”) of total energy in 2002; of this, 13 quads were generated electricity. Should hydrogen become the transportation fuel of choice, as many believe it will—with most hydrogen produced ultimately from fossil fuels—another 12 quads would be required to make hydrogen from water, he wrote.

According to Logan, all the U.S. household wastewater produced in one year contains 0.11 quad organic matter, livestock production waste-water contains 0.3 quad, and food processing wastewater possibly 0.1 quad. Though small, these amounts are potentially significant, says Scott Sklar, the former executive director of the Solar Energy Industries Association and current president of The Stella Group, an energy generation marketing and policy analysis firm. There will be no one-size-fits-all solution to the nation’s energy problems, he says. Instead, energy will come from many sources, many of them small sources, and power will be created through a patchwork of technologies tailored to local circumstances and needs.

MFCs could also become important energy sources in the lesser developed parts of the world, says Logan. These fuel cells used locally produced fuel, and their power output can be managed locally. “Microbial fuel cells [appear] destined, at least at this moment, to utilize some energy resources that are not otherwise available on an industrial scale, like sea bottom sediments, or some biomass from waste,” says Plamen Atanassov, an assistant professor of chemical engineering at the University of New Mexico. One candidate bacterium for MFCs, *Rhodoferax ferrireducens*, was first isolated from sediments collected in Oyster Bay, Virginia; *Geobacter metallireducens* was first isolated from sediments from the Potomac River.

## Breakthroughs Boost Prospects

MFCs go back to the early 1900s, says Angenent. It was at a 1996 American Chemical Society meeting titled “Emerging Technologies in Hazardous Waste Management” that Korean scientists Byung Hong Kim and Doo-Hong Park first described the use of a “mediator-less biofuel cell” to treat wastewater. Breakthroughs in the last five years have suggested fresh promise for this technology.

One breakthrough was the discovery, reported in the 18 January 2002 issue of *Science* by Derek Lovley, a professor in the Department of Microbiology at the University of Massachusetts Amherst, that *Geobacter* produces electricity. That followed the discovery by German and Australian researchers, published in *Bacteriology* in July 1998 (issue 14), that in certain iron-reducing bacteria, the cytochromes—specialized enzymes known to transfer electrons to other proteins—span the outer cell membrane, enabling direct transfer of electrons to external metals and the creation of a circuit. This is the ultimate source of electricity in MFCs. These discoveries opened up the possibility of engineering both the bacteria and the electrodes in the MFC to improve electron transfer.

In the 23 June 2005 issue of *Nature*, Lovley announced the discovery of “nanowires,” literally tiny wires produced by *Geobacter*, which the bacterium presumably uses to transfer electrons. This discovery opened up further possibilities for electron transfer. He also published a study in the Octobe 2003 issue of *Nature Biotechnology* showing that *Rhodoferax* provides a constant flow of electrons while oxidizing glucose at 80% electron efficiency—a boon for drawing power from carbohydrates.

Still another breakthrough was the discovery, published by Park and University of Michigan molecular biologist J. Greg Zeikus in the June 2002 issue of *Applied Microbiology and Biotechnology*, that one could increase power output in MFCs by about sixfold by using mixed microbial communities rather than pure cultures. This is a big advantage for harvesting energy from waste-water, which is microbially diverse, says Angenent. The question of exactly why this is so is an area Angenent plans to address in future research.

The technology has also seen the benefit of engineering advances. A year ago, in unpublished research, Angenent combined the “upflow” system used in methane digesters with the MFC technology to eliminate the need for mechanical pumping and mixing. In the upflow system, wastewater is piped from above the fuel cell, down, around, and then upwards into the bottom of the anode powered by gravity—the opposite of a syphon. Thus, pumping and mixing become unnecessary.

The first microbial fuel cells produced between 1 and 40 milliwatts per square meter (mW/m^2^) of anode electrode surface area, says Logan. In just the past year, he says, his laboratory has generated power in the range of up to 500 mW/m^2^ using domestic waste-water and 1,500 mW/m^2^ with glucose and air. He adds that researchers in Belgium recently achieved 3,600 mW/m^2^ using glucose, although they needed a nonrenewable chemical instead of air for their process.

## Electric versus Hydrogen

MFCs generate electricity, but can be modified to produce hydrogen instead. In both systems, the source of electricity is the chemical energy contained in the bonds of organic compounds. Bacteria, living in biofilms on the anode, break down the organics, separating electrons from protons. These electrons and protons then travel to the cathode, the former via an external wire, the latter by diffusing through the electrolyte, a substance that does not conduct electricity.

In the electricity-generating MFCs, the protons and electrons combine at the cathode with oxygen to form water. This “uses up” the electrons, allowing more to keep flowing from the anode to the cathode.

In the MFC modified to produce hydrogen, the cathode is kept free of oxygen. But in order to make hydrogen, a thermodynamic barrier must be breached. To overcome this barrier, Logan uses a power source to add voltage into the circuit.

The hydrogen MFC appears to be twice as efficient as the electricity-producing cells, says Logan, because in the latter some oxygen leaks back into the anode. However, adding the voltage in the hydrogen-producing system requires about one-sixth of the energy that is produced as hydrogen. Further losses occur if the hydrogen is converted into other forms of energy. Bottom line: in terms of efficiency for electricity as a final product, neither electricity nor hydrogen production possesses a clear advantage.

The main benefit of hydrogen-producing MFCs is that they would provide additional options to fit production to energy needs, says Logan. For example, hydrogen could be stored to make off-peak electricity or for use as a transportation fuel. “But if you just want to use electricity locally, you are probably better off making electricity to start with,” he says.

## Many Technological Challenges

MFC technology is still strictly at the laboratory scale. “[It] doesn’t have its own design principals, and borrows from neighboring technologies,” says Atanassov. “It is absolutely premature to even address [questions of design].”

The cathode oxygen in electricity-producing devices creates a big challenge for MFCs. A “proton exchange membrane” separates anode from cathode, allowing protons to pass, but blocking the larger oxygen molecules from diffusing to the anode. However, some oxygen manages to cross the proton exchange membrane into the anode, where it takes electrons that would otherwise flow in the circuit, reducing the power, says Lovley.

The low power density of MFCs is also a major problem. Researchers working on MFCs measure power density in W/m^2^, while those working on conventional fuel cells measure power density in W/cm^2^, a highly illustrative disparity, says Atanassov. That low power density of MFCs means electrodes—which aren’t cheap—must be exceptionally bulky.

Power density is a function of the interface between the microbes and the electrodes, says Harold Bright, a program manager in the Office of Naval Research, which is funding studies on MFCs. “We have fairly slow electron transfer from the bacteria into the electrode.”

Scale-up for commercial uses adds to the challenges. The current laboratory-scale prototypes use materials that aren’t sturdy enough to be used in a commercial system, such as carbon paper and carbon cloth electrodes. Further, experimental MFCs, now smaller than a beer mug, would need to be as big as a mansion (in large part to compensate for the low power density), undoubtedly greatly increasing the distance between anode and cathode. That, in turn, would slow diffusion of hydrogen from the former to the latter, damping efficiency.

To be competitive with methane digester technology, MFCs’ practical predecessor, the power density must more than double the maximum achieved so far, to 8,500 mW/m^2^, says Angenent. And for this, he says, “another breakthrough is required.”

Advances in microbiology and electrode technology leading to higher rates of electron transfer could improve power density; bacteria could be engineered for better electron transfer. Lovley has been systematically deleting genes for outer membrane cytochromes in order to discern which cytochrome was essential for electricity production. “Now we can determine if engineering *Geobacter* to produce more of this cytochrome and/or modifying the electrode to better interact with the cytochrome will result in more power production,” he says.

There is ample room for improvement. “If *Geobacter* could transfer electrons to electrodes as fast as it can to its natural electron acceptor, ferric iron, the rate of electron flow—that is, the current—could possibly be ten thousand times higher,” says Lovley.

The use of wastes as cost-free substrates will further improve economics, says Logan. Wastes are ideal since their disposal, he says, “is already an economic burden.”

Currently, there is virtually no government funding for MFCs except for use in applications such as remote sensors, which are funded by the Navy, the Department of Energy, and the Defense Advanced Research Projects Agency. “The current laboratory systems that we build cost way too much money for the amount of electricity we get back,” Logan admits. “[But] the same was true of solar energy fifty years ago.” Now solar has become an important—if still small—contributor to the nation’s energy supply, and Logan predicts that MFCs will follow suit.

## Figures and Tables

**Figure:**
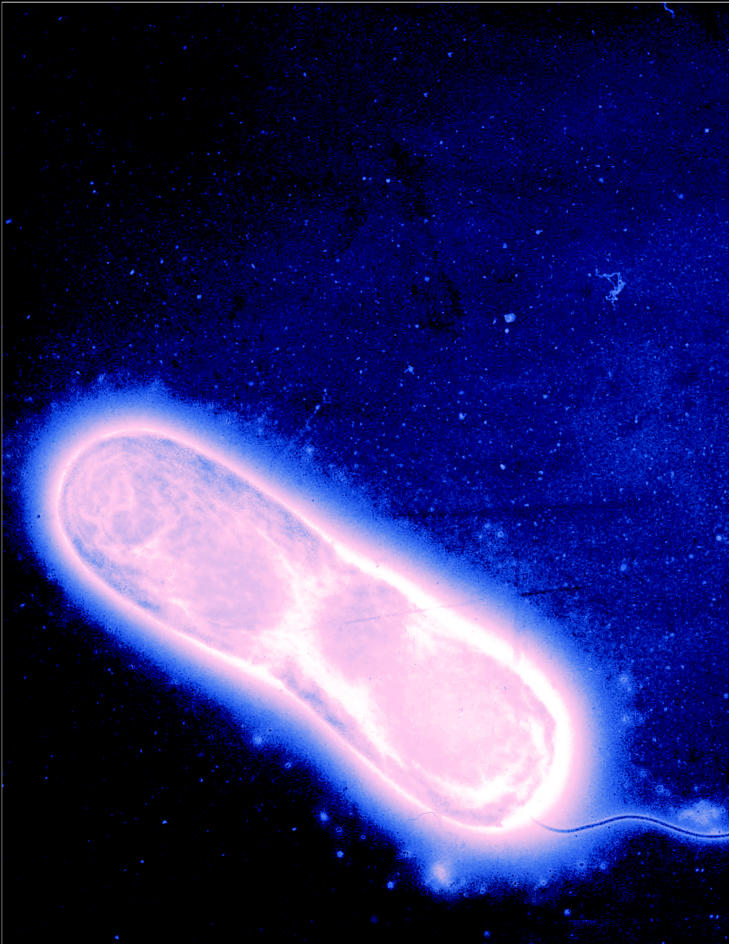


**Figure:**
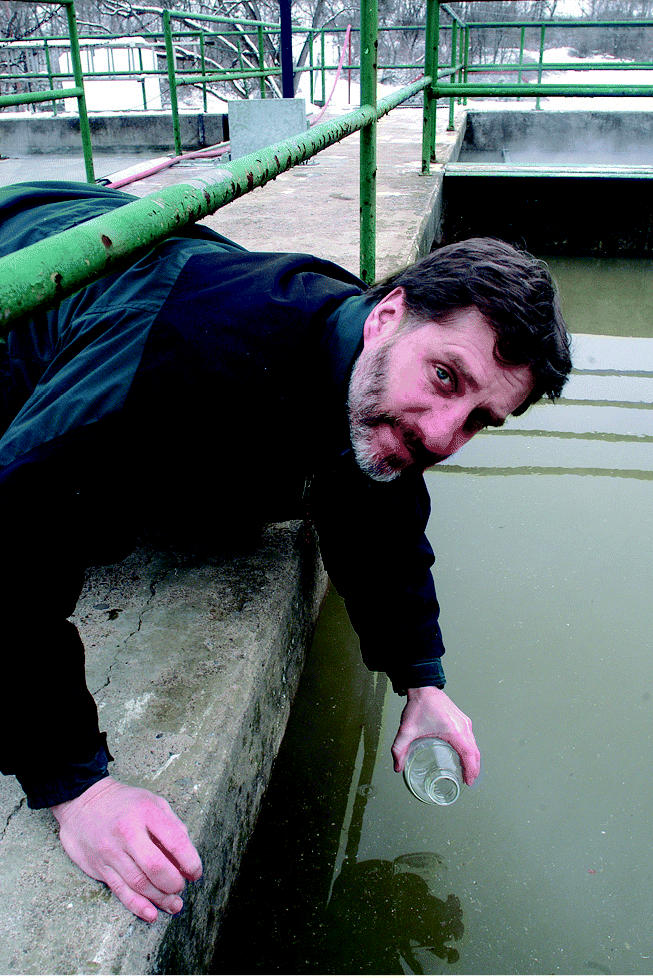
Skimming the surface. Bruce Logan and colleagues at Penn State have begun demonstrating that MFCs can produce electricity directly from wastewater, potentially cutting both power costs and solid wastes.

**Figure:**
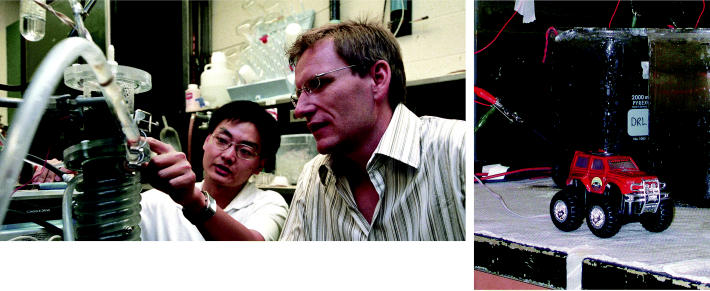
Big plans for small microbes. Jason He (left) and Lars Angenent inspect their MFC. In Derek Lovley’s lab (right), a model SUV is powered by marine geobatteries.

**Figure:**
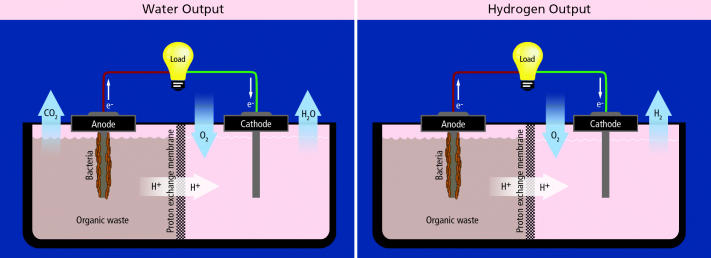
Microbial Fuel Cells: The Basics
